# The Potential Role of Vitamin D in the Pterygium Pathophysiology—A Possible New Therapeutic Perspective and Narrative Review

**DOI:** 10.3390/jcm14113640

**Published:** 2025-05-22

**Authors:** Martina Paradzik Simunovic, Marina Degoricija, Robert Stanic, Janos Terzic, Marko Simunovic

**Affiliations:** 1 Department of Ophthalmology, University Hospital of Split, Spinciceva 1, 21000 Split, Croatia; 2 Laboratory for Cancer Research, School of Medicine, University of Split, Soltanska 2, 21000 Split, Croatia; 3 Department of Medical Chemistry and Biochemistry, School of Medicine, University of Split, 21000 Split, Croatia; 4 Department of Pediatrics, University Hospital of Split, Spinciceva 1, 21000 Split, Croatia; 5 Department of Pediatrics, School of Medicine, University of Split, Soltanska 2, 21000 Split, Croatia

**Keywords:** pterygium, vitamin D, 25-hydroxyvitamin D, ocular surface

## Abstract

Vitamin D plays a vital role in human physiology, including a crucial role in regulating bone metabolism and various extra-skeletal effects. Calcitriol exerts anti-inflammatory effects on monocytes and macrophages by increasing IL-10 production and decreasing the production of proinflammatory IL-1β, IL-6, tumor necrosis factor-α (TNF-α), receptor activator of nuclear factor kappa-Β ligand (RANKL), and cyclo-oxygenase-2 (COX-2). In addition, calcitriol also exerts important effects on adaptive immunity by downregulating MHC-II class and co-stimulatory molecules on antigen-presenting cells, but it also directly affects T lymphocytes. In multiple studies, the influence of vitamin D on eye diseases, including corneal diseases, has been demonstrated. Adequate vitamin D supplementation in patients with dry eye significantly improves tear quality and consequently heals the epithelial cells of the ocular surface. Pterygium is a fibrovascular growth of conjunctival tissue that histologically consists of superficial conjunctival epithelium and an underlying fibrovascular layer. The prevalence of pterygium depends on the region. In zones closer to the equator—“pterygium zone”, it is up to 22%, and outside of them it can be even less than 2%. While UV radiation is recognized as a primary risk factor, other factors, including vitamin D, may influence its development. This review aims to summarize the effects of vitamin D on the pathophysiological mechanism of pterygium and its possible therapeutic impact. Current research suggests that vitamin D is protective through its immunomodulatory and anti-inflammatory properties. Finally, there is still insufficient evidence of the therapeutic benefit of vitamin D in pterygium, and future large-scale randomized controlled studies are needed to elucidate the exact role of vitamin D in pterygium onset and recurrence as well as its potential therapeutic benefit.

## 1. Introduction

Vitamin D plays a crucial role in regulating bone metabolism by maintaining calcium and phosphate homeostasis [1]. The source of vitamin D in humans is nutritional intake and sun exposure [2]. There are two primary forms of vitamin D: cholecalciferol (vitamin D3) and ergocalciferol (vitamin D2). Vitamin D3 is the main source of vitamin D. It can be endogenously synthesized in the skin from cholesterol (7-dehydrocholesterol) with the help of ultraviolet B radiation or exogenously nutritionally ingested (fish, fish oil, mushrooms, eggs, and fortified supplementation). The second source is vitamin D2, which is nutritionally ingested exogenously (plants, yeasts, and mushrooms) [2]. Once in the bloodstream, vitamin D2 and D3 undergo two-step hydroxylation. First step is in the liver, where the activity of vitamin D 25-hydroxylase (encoded by CYP2R1 and CYP27A1) forms 25-hydroxyvitamin D (25-OHD, calcidiol) [2,3]. Calcidiol is released into the plasma bound to the vitamin D-binding protein (DBP), and the second step in activation occurs in the kidneys, where calcidiol is hydroxylated with 25-hydroxyvitamin D-1α hydroxylase (encoded by CYP27B1) to form 1,25-dihydroxyvitamin D (calcitriol), the active form of vitamin D, which can exert its biological functions [2]. Increased expression of the CYP27B1 gene and thus calcitriol production is stimulated by hypocalcemia, hypophosphatemia, parathyroid hormone (PTH), growth hormone, insulin growth factor 1 (IGF-1), and prolactin, while it is dissimulated by hypercalcemia, hyperphosphatemia, high levels of calcitriol, and a bone-derived hormone fibroblast growth factor 23 (FGF23). PTH and FGF23 play a crucial overlapping role in the regulation of synthesis and degradation of calcitriol [4]. In renal proximal tubules, FGF23 binds to the FGFR-αKlotho receptor complex, which activates a signaling cascade that downregulates CYP27B1, thus diminishing the amount of active calcitriol produced [5]. The key role of calcitriol is an increase in intestinal and renal tubular absorption of calcium and phosphates, which promotes bone mineralization and directly affects bone-regulating production of osteocalcin and RANK ligand [2]. It is important to emphasize that there are three types of calcium in plasma: protein-bound, free ionized, and complexed chelated, which includes calcium phosphate, calcium carbonate, and calcium oxalate. Calcitriol-dependent maintenance of physiological levels of calcium primarily depends on free ionized calcium, which is in a complex relationship with phosphate and serum protein levels. In addition to this, FGF23 also induces the transcription of 24-hydroxylase (CYP24A1), which is responsible for vitamin D degradation [6,7,8]. In a feedback manner, calcitriol upregulates the expression of FGF23 in bone osteoclasts by binding to vitamin D response elements (VDREs) [9]. Conversely, PTH is excreted in response to low plasma calcium levels, and it upregulates CYP27B1 expression in kidneys, enhancing intestinal calcium uptake and bone resorption to normalize calcium levels in plasma [10].

After the discovery of vitamin D receptors (VDR) and enzymes for activation of vitamin D in many extra-skeletal tissues, the scientific focus has shifted towards elucidating other effects of vitamin D [11]. Numerous studies have demonstrated that vitamin D is a potent immunomodulator that can reduce inflammation [2,12,13,14]. The anti-inflammatory effect of vitamin D was first demonstrated in the 19th century in patients with tuberculosis who had significantly better survival if they were exposed to sunlight and consumed cod liver [2,14]. This finding was clarified many decades later when it was demonstrated that macrophages and monocytes have increased expression of CYP27B1 upon immune stimulation and produce calcitriol [2,14,15,16]. Calcitriol exerts anti-inflammatory effects on monocytes and macrophages by increasing IL-10 production and decreasing the production of proinflammatory IL-1β, IL-6, tumor necrosis factor-α (TNF-α), receptor activator of nuclear factor kappa-Β ligand (RANKL), and cyclo-oxygenase-2 (COX-2) by targeting mitogen-activated protein kinase (MAPK) phosphatase (MKP)-1 [17,18]. In addition to this, autocrine calcitriol inhibits COX-2 expression in a dose-dependent manner and also stimulates macrophages and monocytes via the VDR receptor to increase secretion of cathelicidin antimicrobial peptide (CAMP) LL-37 [2,14,15,16,19]. LL-37 plays a vital role in antimicrobial defense and mucosal immunity in various human tissues, including the respiratory and gastrointestinal tracts, as well as the skin, oral cavity, and eye [20]. Additionally, LL-37, which is expressed in the corneal and conjunctival epithelium, affects all types of infections, including bacterial and fungal, acting on the destabilization of the mucous membrane, as well as on viruses, damaging the viral envelope and affecting the host cells [2].

Calcitriol also exerts essential effects on adaptive immunity by downregulating MHC-II class and co-stimulatory molecules on antigen-presenting cells, but it also directly affects T lymphocytes [21]. Upon activation, T lymphocytes upregulate VDR expression, calcitriol downregulates Th1, Th17, and Th9 cytokines and upregulates expression of GATA-3 and IL-4 as well as CTLA-4, promoting more tolerogenic Treg suppression [22,23,24,25,26,27]. In addition to this, it was demonstrated that vitamin D induces apoptosis in activated B lymphocytes, suppresses immunoglobulin production, and regulates production of IL-10 and CCR10, an important G protein-coupled chemokine receptor that plays a crucial role in trafficking and retention of lymphocytes in epithelial tissues [28,29,30,31].

Furthermore, in numerous studies so far, the influence of vitamin D on eye diseases, including corneal diseases, has been studied [3]. A recent study by Basaran et al. demonstrated a connection between vitamin D and one of the most common corneal diseases, dry eye [32]. A link between disease severity and the degree of vitamin D deficiency has been demonstrated, as well as significantly higher levels of IL-1β, IL-2, and IL-7 and lower levels of IL-13 in the tear film of patients with lower vitamin D levels [32]. In addition, adequate nutritional intake of vitamin D leads to an increase in 25-OHD levels in tears and aqueous humor, which confirms that systemic levels of vitamin D can affect eye homeostasis and consequent pathophysiological mechanisms in the anterior segment of the eye [33]. Finally, after administration of 1000 units of vitamin D in patients with low 25-OHD levels, there was an improvement in tear quality and consequent healing of the epithelial cells of the ocular surface [34].

Pterygium is characterized as a benign fibrovascular growth of conjunctival tissue that gradually overgrows the cornea [35,36]. Histologically, pterygium consists of superficial conjunctival epithelium and an underlying fibrovascular layer [35,36]. A recent meta-analysis reported a global pterygium prevalence of 12%, with increased incidence in males, the elderly, and the low socioeconomic status group [37]. Additionally, an extensive Korean study involving 9193 subjects over 40 years of age demonstrated a prevalence of 10%, but it is essential to point out that this specific group of elderly subjects may have influenced the overall reproducibility of prevalence in the general population [38]. To fully clarify the prevalence of pterygium and the possibility of overestimation, it is necessary to emphasize that prevalence is significantly higher in regions with geographical latitudes from 40 degrees north and south of the equator. In this area—“pterygium zone”, the prevalence is predicted to be as high as 22%, while outside it is less than 2% [39]. All of the above points to the fact that exposure to ultraviolet radiation (UV) is the main risk factor for the development of pterygium [40,41]. Gradual progression of pterygium initiates the onset of inflammation, leading to visual acuity impairment [36]. The gold standard in treating pterygium remains surgical removal of the fibrovascular growth followed by corrective conjunctival autograft, but unfortunately with a high rate of recurrence [36,42].

This review aims to summarize all the findings about the effect of vitamin D on the insufficiently described pathophysiological mechanism of pterygium formation that could direct future research in the growing and interesting scientific field of vitamin D. A secondary objective of our review is to point out possible therapeutic interventions that might be explored to improve the quality of life of patients and lower the reoccurrence rate of pterygium.

## 2. Pterygium Pathophysiology: Current Insight

Recent meta-analysis demonstrates that the prevalence of pterygium increases in countries close to the equator, which supports previous findings describing a positive correlation of prolonged outdoor activities and consequent sun exposure to the occurrence of pterygium [37,43,44,45]. In relation to this, a significant association was evinced between skin changes caused by increased sun exposure and the occurrence of pterygium [46]. The negative effect of long-term exposure to UV radiation has been described in numerous studies, where it was demonstrated that both UVA and UVB radiation cause DNA damage, consequent impairments in cellular signaling, and the accumulation of oxidative stress [41,46,47]. Human eyes also suffer from numerous negative effects of UV light exposure, including dysfunction of limbal stem cells, induction of inflammation, and a negative impact on stromal fibroblasts [46]. Recent research regarding pterygium pathophysiology focuses on multiple tumor suppressor genes, including protein p53, which is considered one of the key factors in the cascade of pterygium formation [35]. Protein p53 is a tumor suppressor gene, which plays a central role in DNA damage signaling and maintenance of genomic stability [48]. Its negative regulator, mouse double minute 2 (MDM2), downregulates the transcriptional activity of p53, favors the nuclear export of p53, and stimulates its degradation [35,49]. In a study on 48 subjects with pterygium, nuclear export of p53 was demonstrated by immunofluorescent colocalization with MDM2, and the p53 activity was readily restored with MDM2 antagonist in human primary cell culture [50]. Furthermore, increased expression of proto-oncogene B cell lymphoma 2 gene (Bcl-2), an important apoptosis regulator, was observed in recurrent human pterygium tissues [51]. In addition, multiple other factors can influence the formation and progression of pterygium, including various external agents, such as viruses [52]. Viral infections that could affect the onset and progression of pterygium include human papillomavirus, cytomegalovirus, Epstein-Barr virus, and herpes simplex virus [53]. Human papillomavirus is most frequently found in pterygium, but its role in pterygium remains elusive [54,55]. Our recently published data suggest the involvement of microbiota in pterygium development, particularly fungus from the genus Malassezia [56]. Furthermore, mediators of local inflammation, such as various cytokines, growth factors, matrix metalloproteases, and extracellular matrix proteins, were also demonstrated to affect the formation of pterygium [57,58]. In addition to this, a recent study from Kilic et al. demonstrated that systemic inflammation, characterized by increased serum triglycerides and lower HDL in patients with pterygium, might influence pterygium occurrence [59].

## 3. Vitamin D and Pterygium

The link between vitamin D and pterygium has been demonstrated in several studies, which are presented in Table 1, and serum levels of 25-OHD have been reported in most studies [60,61,62,63,64,65,66,67].

In the two largest studies, including the nationwide sample from the Korean National Health and Nutrition Examination Survey, in the period from 2008 to 2012, significantly higher levels of 25-OHD were found in the serum of patients with pterygium compared to controls [62,65]. After the adjustment for additional comorbidities, lifestyle habits and age were in positive association with elevated levels of 25-OHD and the occurrence of pterygium [62,65]. Additionally, this study demonstrated a positive association between elevated levels of 25-OHD and sun exposure with a more severe clinical manifestation and recurrence of pterygium [62,65]. The Korean National Health and Nutrition Examination Survey has been collecting primary health and nutritional data of Koreans since 1998 [68]. The design of the survey primarily was not to study the influence of vitamin D on pterygium, and it is important to emphasize that regardless of the statistically significant difference in vitamin D levels, there is a very small difference between the groups with pterygium and without pterygium in levels of vitamin D. Additionally, all included subjects, including subjects with pterygium, as well as the control group, had mean levels of 25-OHD very close to or below levels for vitamin D deficiency [62,65]. This confirms the thesis that in these studies the primary cause of discretely elevated 25-OHD in the pterygium group was caused by significantly higher exposure to sunlight when compared to the control group, which has previously been proven to be the main factor in the formation of pterygium, rather than a direct influence on the pathophysiological mechanism of pterygium onset and progression. Interestingly, the other three respective studies did not report a significant difference between 25-OHD serum levels in patients with pterygium and the control group, but it should be noted that these studies have a significantly smaller sample size in comparison with South Korean national studies [60,63,64,66]. Conversely, recent studies by Bilak et al. and Sumer et al. demonstrated significantly lower levels of 25-OHD in patients with pterygium in the Turkish population [61,67]. In both studies, the pterygium group had significantly lower 25-OHD values below 20 ng/mL, while the control group had values above 20 ng/mL [61,67]. Additionally, in the study by Bilak et al., the time of vitamin D sampling in the study was within 2 months at the same time of year, and the control group was designed to maximally match the tested group in terms of age, gender, body mass index, and skin color, which minimized other influences on the results [61]. Additionally, pterygium patients also had significantly higher levels of parathyroid hormone in comparison to the control group, which is speculative for secondary hyperparathyroidism that is induced as a consequence of long-term hypovitaminosis D [61]. The authors conclude that excessive UV exposure may cause a decrease in VDR gene expression, as may vitamin D deficiency, and that possible VDR polymorphisms may also reduce the effects of vitamin D on target tissues [61].

In addition, the stated discrepancies in the level of vitamin D in the serum of patients with pterygium may be due to the main limitation of all these studies, which lack a cross-sectional aspect and long-term follow-up of 25-OHD levels in patients. Additionally, considering the long period of recruitment, the seasonal variation of vitamin D levels, and the influence of different skin types, which can be measured using the Fitzpatrick scale, which was used in the selection of the control group in other studies, should be taken into account. It should be emphasized that 25-OHD levels often do not correlate with the highly bioactive form 1,25-dihydroxyvitamin D, and their relationship is very complex and influenced by calcium, phosphorus, and PTH levels [69]. This multi-layered complex relationship in vitamin D metabolism could also have influenced the results of individual studies and the possible reproducibility of the results. Finally, third factors that have affected 25-OHD levels, including sun exposure, could have influenced the results of the studies. Further studies with long-term follow-up of the patients with pterygium are needed to further clarify the effect of vitamin D and include much more detailed and focused research that would investigate the levels of total calcium, ionized calcium, phosphates, PTH, 1,25-dihydroxyvitamin D, and calcitonin.

In a study by Ornek et al., patients with pterygium had significantly higher levels of 25-OHD in tear fluid when compared with the control group, but no association was found between 25-OHD levels and clinical presentation [60]. Recent research on healthy subjects without underlying eye diseases has shown that the level of vitamin D in tear fluid is significantly higher and does not correlate with the level of serum vitamin D levels [70,71]. In addition, in the pediatric population with rhinoconjuctivitis, a significantly higher level of 25-OHD in tear fluid compared to serum was shown, and the authors conclude that there is a possibility that vitamin D affects the pathophysiological mechanism of rhinoconjucitivits [72]. Based on all of the above, future research should focus more on the level of vitamin D in tear fluid and its influence on the formation and progression of pterygium.

Vitamin D receptor is a ligand-dependent nuclear transcription factor that binds to biologically active 1,25 hydroxyvitamin D and regulates the expression of numerous downstream genes [73,74]. Two separate studies have demonstrated differential expression and localization of VDR in epithelial and endothelial cells of patients with pterygium despite unaltered serum 25-OHD levels [64,66]. In the epithelial cells of pterygium patients, VDR was localized both in the nucleus and cytoplasm, which was indicative of increased transcriptional activity [64]. Furthermore, when compared with the control group, significantly higher expression of immunohistochemical staining for VDR was shown in the cytoplasm of the pterygium group, while in the control group, most samples were not positive for nuclear immunoreactivity [64]. Additionally, tissue expression of VDR was upregulated in pterygium intravascular inflammatory cells, endothelial cells of micro-vessels, and subepithelial stroma in comparison to normal conjunctiva [64,66]. Based on their results, the authors concluded that there is a possibility that in pterygium, nuclear VDR represents an alternative pathway through which vitamin D regulates gene expression through its anti-proliferative and anti-inflammatory effects [64].

## 4. Rethinking Pterygium: New Perspectives on Its Pathophysiology and Therapy

Despite numerous studies, the pathogenesis of pterygium formation is still not elucidated. It is important to emphasize that vitamin D possibly plays an important role in the complex cascade of pterygium onset and progression, since it was demonstrated that vitamin D can reduce inflammation and angiogenesis [66]. The immunosuppressive effect of vitamin D has been studied in different parts of the eye, including the cornea, where 1,25-dihydroxyvitamin D altered the expression of IL-1, IL-8, and IL-6 in human corneal epithelial cells after colonization with Pseudomonas aeruginosa [75,76]. Due to the eye’s direct exposure to the environment, the local immunosuppressive effects of vitamin D should be considered as a potential predisposing factor for the involvement of commensal microbiota. In a study by Lu et al., it was demonstrated that 1,25-dihydroxyvitamin D and 24R,25-dihydroxyvitamin D promoted the migration and proliferation of corneal epithelial cells [77]. Furthermore, an in vitro study on the corneal epithelium demonstrated that the cornea expresses 1 α-hydroxylase and 25-hydroxyvitamin D-24-hydroxylase activity, two enzymes crucial for 25-OHD conversion to 1,25-dihydroxyvitamin D and 4R,25-dihydroxyvitamin D, which positively affect the corneal epithelial barrier [78,79]. Several studies on animal models have demonstrated the beneficial effect of topical administration of vitamin D on various diseases of the ocular surface, including post-surgical treatment [80,81]. Similar results were shown in a study on a mouse model where topical application of vitamin D significantly improved corneal injury healing and increased CAMP production was shown, which directly promotes anti-microbial effects [82]. When administered topically, vitamin D can inhibit corneal neovascularization by inhibiting Langerhans cell migration into the cornea in a murine model [83]. The above conclusions are additionally supported by a study on patients with significant hypovitaminosis D with no prior eye pathology that had a significantly lower density of epithelial cells compared to the control group [84]. The beneficial effect of vitamin D on ocular homeostasis was investigated also in a randomized control trial that measured the effectiveness of a topical combination of omega 3, vitamins A and D after phacoemulsification surgery of cataracts, resulting in a reduction in post-surgical corneal staining compared to the control group [80,85]. Moreover, in the study of Jia et al. on pterygium and glaucoma tissues, a positive effect was observed after in vitro application of 1,25-dihydroxyvitamin D on migration, proliferation, and trans-differentiation of human Tenon’s fibroblasts in primary cell culture [86]. All of the above further emphasize the great potential of vitamin D as a possible therapeutic option in preoperative and early postoperative pterygium care. Therefore, we propose future randomized controlled trials that would further clarify the positive effect of topical application of vitamin D on the clinical course of pterygium. Secondly, we suggest that serum 25-OHD levels serve as a screening tool at the onset of pterygium, and as shown, based on an extensive cross-sectional study by Chun et al., higher levels of 25-OHD should be further evaluated as a negative prognostic factor in disease severity and recurrence [62].

In addition to this, our recent findings highlight the possible pro-fibrotic role of macrophages during pterygium progression in response to fungal infection with Malassezia restricta [56]. Vitamin D plays an important role in macrophages, which express VDR and CYP27B1 and can control the bioavailability of the 1,25-dihydroxyvitamin D active form, thereby promoting Th2 and Treg cell responses [87]. 1,25-dihydroxyvitamin D inhibits IFN-γ and IL-17A production and induces a shift from M1 towards M2 macrophages and also upregulates complement receptor immunoglobulin expression in macrophages, thus enhancing phagocytosis of both bacteria and fungi [88,89,90,91,92,93]. Considering these specific immunomodulatory effects of 1,25-dihydroxyvitamin D, future studies should explore the interplay between innate and adaptive immune responses during the onset and progression of pterygium and the preoperative and postoperative vitamin D treatment potential in managing highly recurrent pterygium.

## 5. Conclusions

Studies suggest that vitamin D plays an important role in the pathophysiology of pterygium, exerting an immunomodulatory and anti-inflammatory effect. Studies with a larger sample of different stages of pterygium tissue are needed to further elucidate the exact effect of VDR transcriptional activity on immunomodulation linked to the anti-inflammatory effect of vitamin D in pterygium. New clinical approaches designed to reduce complications after the treatment of pterygium should be further evaluated, including vitamin D administration. Thus, future studies are needed to clarify the effect of vitamin D on different stages of the pterygium. In addition, we would like to propose screening for 25-OHD levels in each new patient with pterygium, especially focusing on vitamin D levels in the tear fluid as a possible new research focus in the pathophysiology of pterygium as a promising target for estimation of the clinical severity and recurrence of the disease. Finally, there is still insufficient evidence of the therapeutic benefit of vitamin D in pterygium, and future large-scale randomized controlled studies are needed to elucidate the exact role of vitamin D in pterygium onset and recurrence as well as its potential therapeutic benefit.

## Figures and Tables

**Table 1:** Clinical studies on levels of 25-hydroxyvitamin D in patients with pterygium in comparison with a control group.

Study	Sample Size *	Gender Female/Male *	Mean Age (yrs) *	25-OHD Levels (ng/mL) *	Additional Findings	Conclusion
Sumer et al. (2025) [67]	100 vs. 60	41/59 vs. 33/27	53.37 ± 5.79 vs. 52.37 ± 5.92	16.25 ± 2.4 vs. 21.1 ± 1.68; *p* < 0.001	Significantly lower levels of vitamin B12, folic acid, and ferritin in the pterygium group.	25-OHD plays a role in the anti-inflammatory effect in pterygium development.
Ornek et al. (2021) [60]	35 vs. 25	14/21 vs. 7/18	51.7 ± 16.7 vs. 50.6 ± 18.7	30.49 ± 8.85 vs. 31.22 ± 9.34; *p* = 0.76	Significantly higher levels of 25-OHD in tear fluid in the pterygium group.	Without a significant difference in levels of 25-OHD.
Bilak et al. (2021) [61]	108 vs. 77	57/51 vs. 47/47	43.31 ± 13.57 vs. 42.28 ± 13.52	12.06 ± 7.07 vs. 20.04 ± 7.96; *p* < 0.001	PTH 42.78 ± 14.18 vs. 29.68 ± 12.31 pg/mL, *p* < 0.001	25-OHD deficiency is possibly a new link in the pathophysiological mechanism of pterygium.
Bilak et al. (2021) [66]	50 vs. 50	29/21 vs. 26/24	51.26 ± 13.27 vs. 51.2 ± 13.9	11.66 ± 5.63 vs. 10.4 ± 6.3; *p* = 0.29	Significantly higher expression of vitamin D receptor in the pterygium group.	Without a significant difference in levels of 25-OHD.
Chun et al. (2019) [62]	869 vs. 11389	461/408 vs. 5776/5613	63.2 ± 0.6 vs. 44.3 ± 0.3	19.7 ± 0.3 vs. 17.4 ± 0.2; *p* < 0.001	Diabetes, MS, hypertension, smoking, and sun exposure are significantly higher in the pterygium group.	Odds for pterygium increase significantly with an increase of 25-OHD and sun exposure.
Kara et al. (2017) [63]	63 vs. 58	33/30 vs. 22/36	58 ± 12 vs. 61 ± 11	15.02 ± 6.73 vs. 14.54 ± 3.57; *p* = 0.731	Male subjects with pterygium had statistically significantly higher levels of 25-OHD.	Without a significant difference in levels of 25-OHD.
Maxia et al. (2017) [64]	41 vs. 47	11/30 vs. 34/13	58.34 ± 15.09 vs. 43.74 ± 11.74	19.47 ± 7.37 vs. 18.22 ± 8.01; *p* > 0.05	Immunohistochemical vitamin D receptor location was different between the pterygium group and healthy controls.	Without a significant difference in levels of 25-OHD.
Jee et al. (2016) [65]	1548 vs. 17630	745/803 vs. 8868/8762	61.7 ± 0.4 vs. 48.9 ± 0.1	20.04 ± 0.2 vs. 18.5 ± 01; *p* < 0.001	Sun exposure is significantly higher in the pterygium group.	Odds for pterygium increase significantly with an increase of 25-OHD even after exclusion of potential confounders.

* Pterygium group vs. control group; 25-OHD, 25 hydroxyvitamin D; PTH, parathyroid hormone; MS, metabolic syndrome.
